# Molecular Cytogenetics in the Era of Chromosomics and Cytogenomic Approaches

**DOI:** 10.3389/fgene.2021.720507

**Published:** 2021-10-13

**Authors:** Thomas Liehr

**Affiliations:** Jena University Hospital, Institute of Human Genetics, Friedrich Schiller University, Jena, Germany

**Keywords:** cytogenomics, chromosomics, topologically associating domains (TADs), copy number variations (CNVs), small supernumerary marker chromosomes (sSMCs), chromosomal heteromorphisms (CHMs), glass-needle based chromosome microdissection (midi), chromothripsis

## Abstract

Here the role of molecular cytogenetics in the context of yet available all other cytogenomic approaches is discussed. A short introduction how cytogenetics and molecular cytogenetics were established is followed by technical aspects of fluorescence *in situ* hybridization (FISH). The latter contains the methodology itself, the types of probe- and target-DNA, as well as probe sets. The main part deals with examples of modern FISH-applications, highlighting unique possibilities of the approach, like the possibility to study individual cells and even individual chromosomes. Different variants of FISH can be used to retrieve information on genomes from (almost) base pair to whole genomic level, as besides only second and third generation sequencing approaches can do. Here especially highlighted variations of FISH are molecular combing, chromosome orientation-FISH (CO-FISH), telomere-FISH, parental origin determination FISH (POD-FISH), FISH to resolve the nuclear architecture, multicolor-FISH (mFISH) approaches, among other applied in chromoanagenesis studies, Comet-FISH, and CRISPR-mediated FISH-applications. Overall, molecular cytogenetics is far from being outdated and actively involved in up-to-date diagnostics and research.

## Introduction

This review is about “molecular cytogenetics” including 1) the historical perspective of its development from cytogenetics, 2) technical aspects, 3) available probe sets, and 4) variants and applications of the basic fluorescence *in situ* hybridization (FISH) approach. According to present zeitgeist, it is trendy to replace the word cytogenetics and/or the application of whole genome oriented molecular genetic approaches, by the designation “cytogenomics”. Thus, first a few comments on this point are necessary to understand why a change from the designation “molecular cytogenetics” to “molecular cytogenomics” is not justified by any means, even though “molecular cytogenetics” is clearly a “cytogenomic approach”.

### Cytogenomics and Chromosomics

In literature, the neologism “cytogenomics” reflects normally only “the changes in technology under its purview” (McGowan-Jordan et al., 2020), which is overall an a bit weak argument to replace the long standing, clearly defined word “cytogenetics” by a new one. Strikingly, a definition for this word coined already in 1999 (Bernheim, 1999) is hard to find in the literature; in 2019, it was referred to as “a general term that encompasses conventional, as well as molecular cytogenetics (FISH, microarrays) and molecular-based techniques” (Siva et al., 2019). It is here suggested that the word “cytogenomics” should rather be used with the goal to paraphrase a new field of research in genomics and diagnostics in human genetics, with an integrative and comprehensive view. Cytogenomics is, under this definition, nothing else than an equivalent wording for “chromosomics”, a designation introduced in 2005 by Prof. Uwe Claussen (Jena, Germany) (Claussen, 2005; Liehr, 2019). He suggested to introduce the term chromosomics being equal to cytogenomics to bring the three-dimensional morphologically of chromosomes into the focus of research, as this is essential for gene regulation. Under this generic term, all chromosome-related studies should be summarized to introduce novel ideas and concepts in biology and medicine, thus having an integrative effect on the field. The latter is an extraordinary thinking approach, as in most other cases a new “omics”-field was introduced to separate the corresponding field from all the others (Bernheim, 1999; Claussen, 2005).

## Cytogenetics—Historical Aspects

Cytogenetics is the study of chromosomes, which were seen first in 1879; Walter Flemming was at that time the one to introduce the designations “chromatin” and “mitosis” (Flemming, 1879). “Molecular cytogenetics” developed from cytogenetics field, later. In 1888, Heinrich W. Waldeyer introduced the name “stained body” as “chromosome” (Waldeyer, 1888) for what Gregor Mendel already postulated as “Kopplungsgruppen”, which refers to “linked up groups” in German (Mendel, 1866). Walter Sutton and Theodor Boveri were then the first to suggest in 1902/03 the chromosome-theory of inheritance (Boveri, 1902; Sutton, 1903).

Human cytogenetic discipline, in particular, underwent different developmental steps—each providing more and better possibilities for the characterization of acquired and constitutional chromosomal aberrations. Reliable identification of such alterations started with banding cytogenetics technique, introduced by Dr. Lore Zech (Uppsala, Sweden) by 1970 (Schlegelberger, 2013). Further approaches, as C-banding (Arrighi and Hsu, 1971) and silver staining of nucleolus organizing (Goodpasture and Bloom, 1975), complemented the cytogenetic method-set by mid to end of the 1970s. GTG-banding (G-bands by Trypsin using Giemsa) (Seabright, 1971) is still considered as the gold standard of chromosomal diagnostics (Schlegelberger, 2013). Even though, without any proof of evidence, cytogenetics is called dead for decades (Salman et al., 2004), it is imperative to remember that each single available “cytogenomic approach” provides unique and complementary possibilities to obtain information from a genome; the latter can be retrieved at single cell-, or millions of cell-level and at different resolutions (Hochstenbach et al., 2019). Cytogenetics has a low resolution of 5–10 mega base pairs, but enables a whole genomic view; it is cost-efficient and single cell oriented; i.e., it is able to pick up small mosaics. Retrospectively one can state that molecular cytogenetics was developed with the following goals: 1) to take still advantage of possibilities of banding cytogenetics, but 2) to overcome the limitation of its low resolution, and 3) to include the possibility to analyze interphase cells, too (Zhang et al., 2018). Between 1969 and 1986, *in situ* hybridization (ISH) could exclusively be performed as a radioactive variant. Nonradioactive probe labeling using biotin as nonradioactive hapten (detectable by fluorochrome-coupled avidin) was developed in 1981, and thus, not earlier than in 1986, the first fluorescence ISH (FISH) on human chromosomes was reported. Besides FISH, also the primed *in situ* hybridization (PRINS) technique was an important molecular cytogenetic approach between 1989 and 2010 (Koch et al., 1995).

## Molecular Cytogenetics—FISH

FISH, the only remaining approach of molecular cytogenetics, was first available as single (Pinkel et al., 1986) and dual color approach (Hopman et al., 1986); since 1998, it could also be applied in multicolor FISH (Nederlof et al., 1998). The first mile stone in multicolor-FISH was the simultaneous use of all 24 human whole chromosome paints in one experiment (Speicher et al., 1996; Schröck et al., 1996). Besides many other multicolor-FISH (mFISH), probe sets were developed and are summarized elsewhere (Liehr, 2021). FISH is used in multiple ways in diagnostics and research—one of the latest and most interesting developments for both fields maybe at present the molecular combing approach (Florian et al., 2019).

**FIGURE 1:**
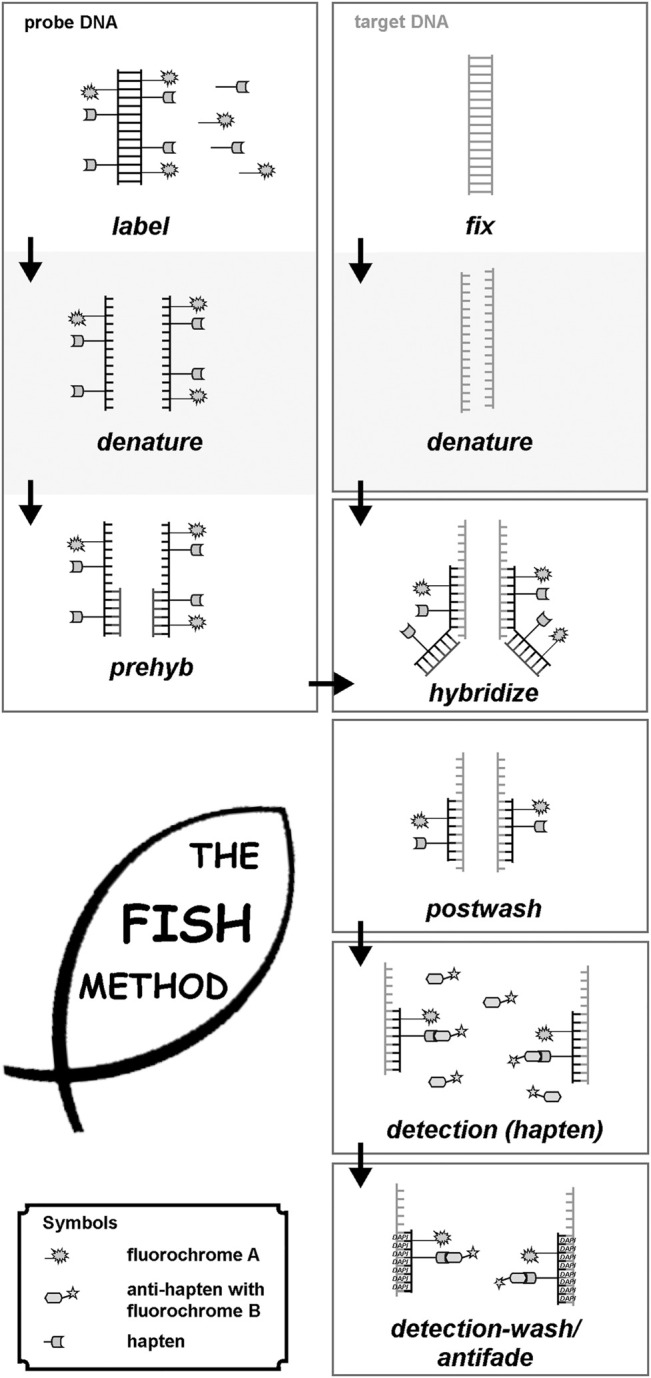
Principle of FISH is given here schematically. First probe- and target DNA are denatured. Probe-DNA is either labeled (commercial probes), or needs to be labeled, e.g., by PCR-approaches. Probe-DNA is pre-hybridized with unlabeled repetitive DNA and then hybridized to the target DNA, being fixed on a glass-slide. After hybridization, postwashing is done to get rid of superfluous probe and blocking DNA. In case a non-fluorescent hapten was used to label the probe-DNA, this has to be detected by an anti-hapten with a fluorochrome. Finally, after washing of the detection-solution, slides can be sealed with antifade and DNA-staining dye (like DAPI = Dipehnylaminoindol), and evaluated under a suited microscope.

The principle of FISH is simple (Pinkel et al., 1986), and nowadays, it is a well-established approach with hundreds of commercially available and applicable probes and probe sets (Liehr, 2017; 2021) (Figure 1). Nonetheless, to find the right laboratory protocols needed initially 1 decade.

Molecular cytogenetics can be performed on different kinds of samples. While in banding cytogenetics it is imperative to have a chromosomal preparation, FISH can be done also on tissue sections and in interphase nuclei. Necessary are always a target-DNA (metaphases or interphases, or for molecular combing (see below) DNA-fibers) and a probe-DNA. The latter has to be labelled with an under a fluorescence microscope detectable hapten (see below). The following steps have to be performed (see also Figure 1):

• Denaturation of target- and probe-DNA;
• Incubation of target- and probe-DNA in a hybridization solution at 37°C for several hours (with or without blocking of repetitive DNA-sequences to avoid possible background);
• Washing off superfluous probe-DNA with suited buffers;
• If necessary, detection of the hapten bound to probe-DNA with a fluorophore-labelled antibody; otherwise—if probe-DNA is already fluorescence labelled—addition of antifade-solution and coverslip;
• Evaluation under the fluorescence microscope.
• More technical details can be found elsewhere (Liehr, 2017; 2021).

### Types of Target-DNA/Samples in FISH

For FISH experiments, samples need to contain intact, non-degraded high molecular weight target-DNA. All tissues of any species fulfilling this prerequisite can be applied in FISH (Liehr, 2017)—even bacteria can be accessed (Bottari et al., 2006). Accordingly, in multicellular organisms, native cells, extracted nuclei, tissue sections, metaphase chromosomes, or pure DNA can be used as target-DNA. In human, most often used are easily accessible tissues, or such acquired during surgeries, e.g., peripheral blood lymphocytes, bone marrow cells, skin fibroblasts, buccal mucosa, hair root cells, urine derived cells, amniotic fluid, chorion biopsy derived cells, gametes (sperm and oocytes), or tumor cells (including formalin-fixed paraffin-embedded tissues). More details can be found elsewhere (Liehr, 2017; 2021).

### Types of Probe-DNA Suited for FISH

On the one hand, there are commercially available probes, especially for molecular cytogenetics based chromosomic research and diagnostics in humans. These probes are usually ready to use and labeled with corresponding fluorophores or non-fluorescent haptens (for review on commercial probes for cancer cytogenetics (Liehr et al., 2015)). The second type of probe-DNA for FISH are in house probes, which need to be labeled, either by PCR-based approaches, Nick-translation, or the so-called Universal Linkage System (ULS) (Liehr, 2017; 2021). In the following, five basic types of probe-DNA applied for FISH are listed.

#### Locus Specific Probes

Locus-specific probes (LSPs) are normally derived from molecular cloning experiments. Accordingly, genetic vectors, including all kinds of plasmids, bacterial and yeast artificial chromosomes, or others are suited if they contain the wanted insert of species-specific DNA to be targeted by FISH, with inserts of a minimal size of 12 kb (Liehr, 2017; 2021). Alternatively, also contiguous probes may be used (Smith et al., 1997), or for mapping purposes, even smaller single copy probes (Nguyen et al., 2019).

#### Repetitive Probes

Repetitive DNA can be easily visualized in FISH experiments. Thus, repetitive probes, targeting centromeres, telomeres, or other repetitive, e.g., interspersed satellite-DNAs, result in strong and easily evaluable signals. Interestingly, at least one repetitive DNA (D4Z4) localized in 4q24 has some meaning in human genetic diagnostics and can be traced by molecular combing (Nguyen et al., 2019).

#### Partial Chromosome Paints

Partial chromosome paints (pcps) can be established by glass needle-based chromosome microdissection (midi) (Al-Rikabi et al., 2019). Pcps simultaneously stain at least 1 or 2 euchromatic chromosomal subbands and are normally not larger than a chromosome arm.

#### Whole Chromosome Paints

A whole chromosome paint (wcp), staining an entire chromosome can either be established by midi (Ferguson-Smith et al., 2005) or by chromosome flow sorting (Sabile et al., 1997). Besides, interspecies hybrids (e.g., mouse/human somatic cell hybrid) have been used as sources of species-specific wcp probes (Sabile et al., 1997).

#### Whole Genome Probes

Even whole genomic DNA can be applied in FISH. This only is informative when using a trick: in a comparative genomic hybridization (CGH) setting (Kallioniemi et al., 1992), two whole genomes labelled in two different colors are co-hybridized to normal human blood-derived metaphases. This approach can be used as CGH in comparative cytogenomics in evolution-research (Majka et al., 2017), or as molecular karyotyping or array-CGH (aCGH) in human genetic diagnostics (Pinkel et al., 1998).

#### Molecular Cytogenetic Probe Sets

It is possible to combine the just listed different FISH-probes in two-to multicolor-FISH probe sets (Liehr, 2017; 2021). As it is impossible to list all of yet done combinations, in the following sections, only some probe sets together with their applications in the concert of cytogenomic approaches are included. Their impact on chromosomic research and human genetic diagnostics is discussed, too.

## Molecular Cytogenetics in Routine Diagnostics

Even though there are (elsewhere (Liehr, 2020) in more detail discussed) problems of getting sufficient reimbursement for routine FISH-diagnostics, molecular cytogenetic is and remains of constant, and even growing importance in many fields of genetics. Fields of applications include pre- and postnatal as well as tumor diagnostics on cytogenetically worked up cells, with interphase-, as well as metaphase-FISH being performed. Also, FISH is routinely done in FFPE (formalin fixed, paraffin embedded) material for solid tumor diagnostics in pathology (Liehr, 2017).

All aforementioned probes combined in two-to multi-color-FISH approaches are applied in molecular cytogenetic routine diagnostics. While in metaphase-FISH there is no restriction in number and types of probes, in interphase-FISH preferentially less than six LSPs and/or centromeric probes are applied. Especially during last decade, probe sets were developed not only to detect loss or gain of copy numbers, reciprocal translocations/gene fusions or gene splitting, but also such to distinguish different fusion partners and/or detect even inversions in interphase nuclei (Liehr, 2017).

Diagnostic applications of molecular cytogenetics are already summarized elsewhere, and thus not further treated here; corresponding literature is listed in Table 1.

**TABLE 1:** Literature and reviews on molecular cytogenetics in routine diagnostics for the major fields of application in human genetic diagnostics.

Routine diagnostics	References
Prenatal FISH	Weise and Liehr (2008), Pellestor et al. (2011), Liehr (2017), chapter “Commercial FISH-probes” Sala et al. (2019)
Postnatal FISH	Liehr (2017), chapter “Commercial FISH-probes” Liehr and Hamid Al-Rikabi (2018)
Tumor cytogenetic FISH in leukemia and lymphoma	Liehr et al. (2015), Liehr (2017), chapter “Commercial FISH-probes” Cui et al. (2016)
FISH in solid tumors	Cheng et al. (2017), Liehr (2017), chapter “Commercial FISH-probes” Liehr (2017), “interphase FISH in diagnostics”

## Molecular Cytogenetic Applications for Chromosomic Research in the Concert of Cytogenomics Approaches

**FIGURE 2:**
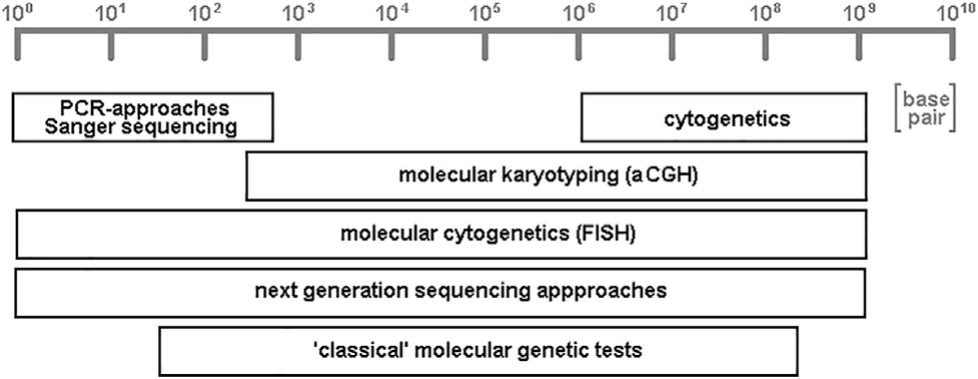
Schematic depiction of resolutions achievable by variants of different cytogenomic approaches. Cytogenetics provides low resolution, but accesses the single cell level; PCR- and Sanger sequencing enable unique high resolution of genomes, while “classical” molecular genetic tests (like Southern-blot and restriction fragment length polymorphism analyses) aCGH (array-comparative genomic hybridization) have low to intermediate resolution. Molecular cytogenetics, together with its variant molecular combing, and all variants of next-generation sequencing approaches are the only two general techniques which can access the whole genome, from low to high resolution.

Some of the approaches listed below are able to help in characterization of DNA-stretches of several to hundreds of base pair in length, while others are directed towards chromosomal subregions, bands, or whole chromosomes; some even give information on whole genome level. It must be admitted that here a subjective selection of research and diagnostic fields has been put together. This is necessary due to the sheer amount of the possible FISH-applications, and influenced by the focus of the author in human genetics field. Completely not covered are, e.g., molecular cytogenetic applications in plant-research (Lavania, 1998; Liehr, 2017) or microorganisms (Bensimon et al., 1994; Liehr, 2017). In the given examples, it will be highlighted that molecular cytogenetics (like next-generation sequencing approaches; Figure 2) is one of two cytogenomic approaches being able to analyze whole genomes from base pair to chromosomal levels.

For limitations of FISH in general, there are to mention, that 1) the resolution in standard FISH is limited to kilo-to megabasepair (except for molecular combing approach), and thus exact mapping of chromosomal breakpoints needs combined approaches like, e.g., applied in Jancuskova et al. (2013) or Moysés-Oliveira et al. (2019), 2) disease causing gene mutations at base pair level normally cannot be accessed by FISH—one exception was recently published (*Molecular Combing* Section) (Nguyen et al., 2019), 3) as in cytogenetics and next-generation sequencing (NGS) for qualified evaluation and interpretation of FISH-results experienced specialists are needed, 4) corresponding to question to be studied costs of consumables may be relatively high, and 5) number of probes being applied simultaneously is limited by number of fluorochromes and software; however, recent developments proved also solutions for this (Su et al., 2020).

### Molecular Combing

The approach molecular combing refers to the physical combing of high molecular weight DNA on a glass surface. This approach was already suggested in 1994 and was deduced from what others published as fiber-FISH (Heng et al., 1992). However, molecular combing got a boost during last few years, as then molecular combing became also commercially available (Florian et al., 2019). This approach enables research on most stretched DNA-fibers; FISH probes can be hybridized and basic studies on DNA-replication, replication kinetics, but also for copy number variations of satellite sequences down to single nucleotide polymorphisms (SNPs) are possible and can be visualized (Florian et al., 2019). Besides, diagnostics for facio scapulohumeral muscular dystrophy (FSHD) became much more feasible, as by molecular combing the D4Z4 sequence in 4q35 and 10q26 can be clearly distinguished from each other (Nguyen et al., 2019). Much more breakthroughs from this high resolution FISH-approach are to be expected.

### Chromosome Orientation-FISH (CO-FISH)

Chromosome orientation-FISH (CO-FISH) enables to selectively mark exactly one of the two homologous DNA strands of a chromosome. This is done by incorporation of 5-bromodeoxyuridine (BrdU) in one DNA strand and destroying it by UV-light and *EXOIII* enzyme treatment (the latter detects UV-induced gaps and starts degradation of DNA strand there) (Goodwin and Meyne, 1993). CO-FISH has been successfully applied to study orientation of repeated sequences or long unique DNA sequences by now (Liehr, 2017). The unique possibilities and advantages of this specialized FISH-approach have not been explored in full by scientific community, yet.

### Telomeres Accessed by Q-FISH

Telomeres are important objects of research, as they are on the one hand known to be important for chromosome stability and also suggested to play a role in aging, cancer development but also apoptosis and senescence (Smith et al., 2019). Telomeres are low-copy repetitive elements, which are hard to access by molecular genetic approaches like sequencing. Telomere length can only be measured by few approaches. Available assays include 1) quantitative polymerase chain reaction, 2) terminal restriction fragment analysis, 3) telomere dysfunctional induced foci analysis, 4) single telomere length analysis, 5) telomere shortest length assay, and 6) quantitative FISH (Q-FISH). The latter is the only available *in situ* approach (Lai et al., 2018). It is even principally possible to do chromosome-specific telomere length studies by that technique.

### Parental Origin Determination FISH (POD-FISH)

In 2001, the now well-known copy number variations (CNVs) were reported first for the human genome (Redon et al., 2006). Their detection was due to the, during that time in large scale studies applied approach aCGH—nowadays mostly referred to as CMA (chromosomal micro-array). CNVs, these previously undetectable structural variations of the human genome comprise losses, gains, insertions, and inversions in kilo-to mega-base-pair-range. CNVs of that size are accessible by FISH. Thus, it was logical to develop the following idea: these CNVs have an individual pattern along each chromosome and it is possible to use them, as before microsatellite markers, as markers to distinguish individual homologous chromosomes. When studying a trio (father, mother, and child) by microsatellite analyses, it is possible to follow up inheritance of chromosomes. Taking advantage of CNVs, the same can be done in trio-analyses of chromosome-preparations. Apart from uniparental disomy testing, by this approach (called parental origin determination FISH - POD-FISH) also the inheritance of individual chromosomes can be visualized. In microsatellite analyses, this is not possible as no individual chromosomes can be distinguished (Liehr, 2017; Weise et al., 2015).

### Inter- and Intrachromosomal Interactions

The spatial organization of chromosomes in interphase nuclei, as well as the organization of metaphase chromosomes—which turned out to be not that different—is, as we know now, key to understand gene regulation (Daban, 2020). Inter- and intrachromosomal interactions can be studied in two ways: On the one hand, there are the high-throughput chromosome conformation capture (also abbreviated as high 3C or Hi-C) approaches, used for genome architecture mapping providing a multi-cell based genomic view. Such high-throughput, sequencing-based approaches have provided tremendously to our knowledge of genomic architecture, by giving contact information chromatin loci pairs. However, real 3D position information of individual alleles and/or loci cannot be deduced from this kind of data (Su et al., 2020). On the other hand, the three-dimensional genome organization can be studied on single cell level either by single-cell Hi-C or by imaging-based approaches. The latter enable spatial positioning of several chromatin loci at a time in single cells. Specifically, it is the FISH approach, which provides such characterizations in fixed cells. Living cells can since recently accessed by the clustered regularly interspaced short palindromic repeats (CRISPR) system (Su et al., 2020). Topologically associating domains (TADs) (Dixon et al., 2015) and related intra- and inter-chromosomal interactions (Maas et al., 2019) were recently identified by combining both complementing approaches. Especially here molecular cytogenetics is an indispensable research tool.

### Multicolor-FISH in Research

**FIGURE 3:**
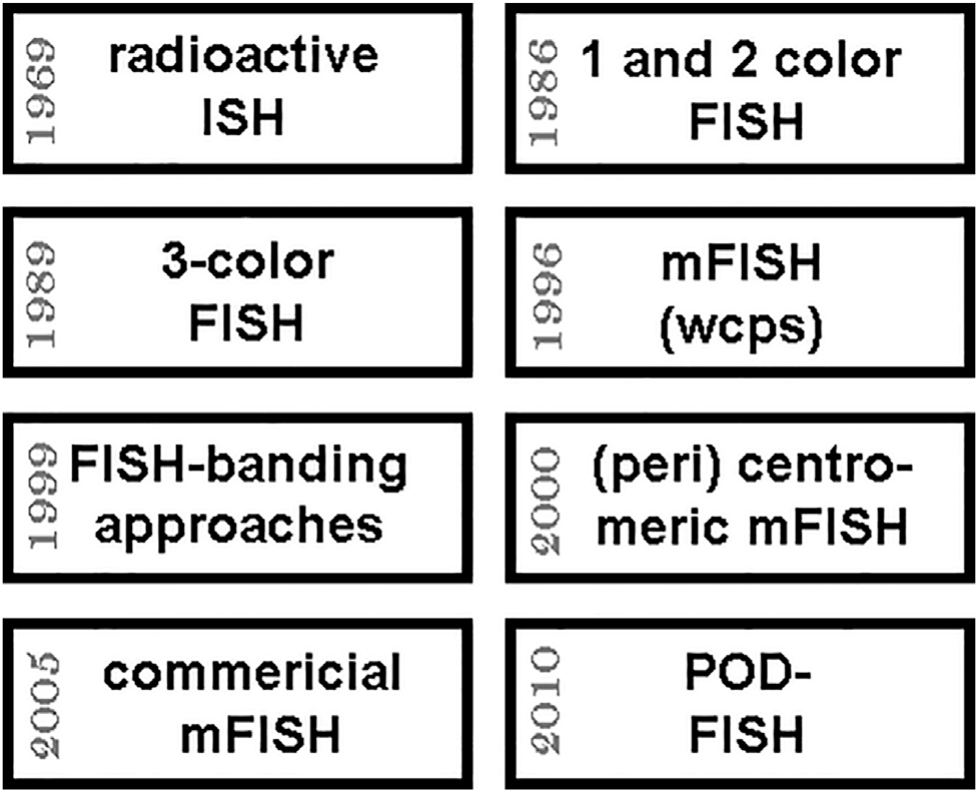
Approximate timeline of important steps towards multicolor FISH (mFISH) is shown in the upper 4 boxes; the lower boxes give four further years when important mFISH-applications were kicked-off and/or became more and more available to research and diagnostics. Abbreviations: ISH = *in situ* hybridization; POD-FISH = parental origin determination FISH (see 4.4.); wcps = whole chromosome paints.

Multicolor-FISH (mFISH) approaches and probe sets are applied—if reimbursed by the health systems somehow (Liehr, 2020) —in routine diagnostics and often independent of such issues in research; for review, see (Liehr, 2017; 2021). As mentioned under point 3, mFISH routine application in human genetics was initiated in 1996 by simultaneous painting of all 24 human chromosomes by whole chromosome probes applied in multiplex-FISH (M-FISH) and spectral karyotyping (SKY). Afterwards, countless mFISH assays have been established (Liehr, 2017; 2021). While most mFISH-probe sets for the characterization of the human genome were implemented primarily to study acquired or inherited chromosomal aberrations in diagnostics (see also point 4.7. below; Figure 3), others are pure research oriented. Specifically of interest are here the FISH-based chromosome-banding approaches (FISH-banding), like multicolor banding. Murine multicolor banding (mcb), for example, is used in studies in murine chromosome evolution or to characterize murine tumor cell lines (Liehr, 2021).

### Research on Small Supernumerary Marker Chromosomes

**FIGURE 4:**
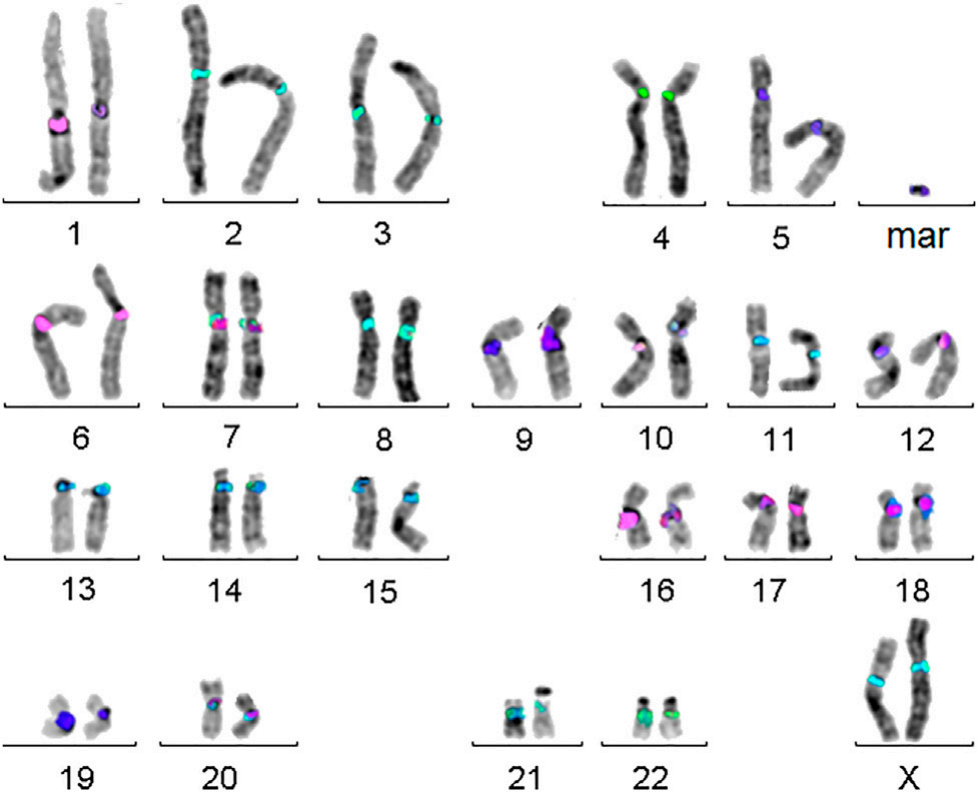
Centromere-specific multicolor-FISH (cenM-FISH) was established to characterize small supernumerary marker chromosomes (sSMCs) for their chromosomal origin. As majority of sSMCs is derived from pericentric regions, an mFISH probe set consisting of probes specific for centromeres of chromosomes 1, 1/5/19, 2, 3, 4, 5/6, 7, 8, 9, 10, 11, 12, 13/21, 14/22, 15, 16, 17, 18, 20, 22, X, and Y is suited to characterize the here shown sSMC as a being derived from a chromosome 5.

Small supernumerary marker chromosomes (sSMCs) are a rare condition in human, resembling B-chromosomes in many other species. They can be found in ∼3.3 million carriers worldwide, with ∼2.2 million of them being asymptomatic. The remainders constitute a pool of patients with dozens of rare diseases. As also clinically normal sSMC carriers can have partial tri- or tetrasomies of euchromatic centromere–near regions they are as well in focus of research [for review (Liehr, 2021)]. As recently shown, molecular cytogenetics is the most straightforward approach to characterize sSMCs for their origin and genetic content, as sSMCs tend to be missed by molecular karyotyping or sequencing approaches due to their (low) mosaic and/or heterochromatic state (Liehr, 2021). The best suited approach to characterize sSMC’s origin is the so-called centromere-specific multicolor-FISH (cenM-FISH) (Liehr, 2021)—an example for an sSMC derived from chromosome 5 characterized by cenM-FISH is shown in Figure 4.

### Chromoanagenesis—Research

Complex chromosomal rearrangements and how they form has been studied for decades, applying cytogenetics and molecular cytogenetics (Heng et al., 2020). Besides, (molecular) cytogenetic studies already reported phenomena like single cells with extremely rearranged chromosomes and/or chromosome pulverization (Stephens et al., 2011). However, those results were widely ignored until they were “newly discovered” as chromothripsis in 2011 based on NGS studies (Stephens et al., 2011). Since then phenomena like chromothripsis, chromoanasynthesis and chromoplexy were subsumed under the term chromoanagenesis (for review see (Hattori and Fukami, 2020)). Meanwhile, there are more and more chromoanagenesis studies combining advantages of NGS and molecular cytogenetics (e.g., (Gu et al., 2016)).

### Chromosomal Heteromorphisms and Repetitive DNA-Elements

Chromosomal heteromorphisms (CHMs), like length variants of acrocentric’s short arms, are still exclusively accessible by cytogenetics and can be characterized in more detail only by molecular cytogenetics. These CHMs, consisting mainly of repetitive DNA-elements, like satellite DNAs, are definitely understudied. These genomic regions are widely ignored, and this is maybe best underlined by the fact that all in the 1980s characterized satellite DNAs known to be localized in the pericentric and/or heterochromatic regions of the human chromosomes are yet not included in any genomic browser. Their localizations and sizes are published, the probes like DXZ1 and DYZ3 are commercially available centromere-specific probes for chromosomes X and Y, and however, they remain unmentioned in the human genome browsers (Liehr, 2021).

### FISH and Microdissection

Another underrated cytogenomic possibility is the application of glass-needle based chromosome microdissection (midi) for research (Maslova et al., 2015). Here, picogram of DNA can be taken directly from chromosomes and studied in multiple ways afterwards, including NGS approaches and others. Also prior FISH-labelled metaphases can be applied in midi, which can help to extract the correct (part of a) chromosome (Kosyakova et al., 2013).

### Comet-FISH

Comet-assay is also a longstanding approach, leading to a bunch of new research possibilities if combined with molecular cytogenetics. “The comet assay is a rapid and very sensitive fluorescent microscopy-based method for measuring DNA damage, protection, and repair at the level of individual cells. In this assay, cells are embedded in agarose, lysed, and then electrophoresed. Negatively charged broken DNA strands exit from the lysed cell under the electric field and form a comet with “head” and “tail”. The amount of DNA in the tail, relative to the head, is proportional to the amount of strand breaks. Results from the comet assay alone reflect only the level of overall DNA damage in single cells. The introduction of FISH in comet has allowed adding new abilities and to enhance resolution and validity of these two methods. FISH permitted to supplement potential of the comet assay with an opportunity to recognize genome regions of interest on comet images. The use of Comet-FISH will enable to achieve a higher sensitivity for the adequate hazard assessment of mutagens and will lead to a better understanding of the biological mechanisms involved” (Hovhannisyan, 201).

### CRISPR-Mediated FISH-Applications

As already seen, molecular cytogenetics can be combined with multiple other approaches, which can lead to new possibilities to decipher multiple biological questions. The most recent advance is to combine FISH with the CRISPR/Cas9 system (Carroll, 2012); this can be done to get FISH-results in dead cells (Němečková et al., 2019), as well as to perform CRISPR-mediated live imaging, the latter allowing insights into living cells (Anton et al., 2014). Which new chromosomic research will become possible by these approaches has to be waited for.

## Conclusion

Overall, it is still valid what Serakinci and Koelvraa, 2009 stated in 2009: “FISH techniques were originally developed as extra tools in attempts to map genes and a number of advances were achieved with this new technique. However, it soon became apparent that the FISH concept offered promising possibilities also in a number of other areas in biology and its use spread into new areas of research and also into the area of clinical diagnosis. In very general terms the virtues of FISH are in two areas of biology, namely genome characterization and cellular organization, function and diversity. To what extend FISH technology will be further developed and applied in new areas of research in the future remains to be seen” (Serakinci and Koelvraa, 2009).
